# BLAST-XYPlot Viewer: A Tool for Performing BLAST in Whole-Genome Sequenced Bacteria/Archaea and Visualize Whole Results Simultaneously

**DOI:** 10.1534/g3.118.200220

**Published:** 2018-05-22

**Authors:** Yagul Pedraza-Pérez, Rodrigo Alberto Cuevas-Vede, Ángel Bernardo Canto-Gómez, Liliana López-Pliego, Rosa María Gutiérrez-Ríos, Ismael Hernández-Lucas, Gustavo Rubín-Linares, Ygnacio Martínez-Laguna, Jesús Francisco López-Olguín, Luis Ernesto Fuentes-Ramírez

**Affiliations:** * Instituto de Ciencias; † Facultad de Ciencias de la Computación, Benemérita Universidad Autónoma de Puebla, Puebla, Pue., México CP 72570; ‡ Instituto de Biotecnología, Universidad Nacional Autónoma de México, Cuernavaca, Mor., México CP 62210

**Keywords:** large scale BLAST, bacterial genomes, operon search, BLAST-XYPlot viewer

## Abstract

One of the most commonly used tools to compare protein or DNA sequences against databases is BLAST. We introduce a web tool that allows the performance of BLAST-searches of protein/DNA sequences in whole-genome sequenced bacteria/archaea, and displays a large amount of BLAST-results simultaneously. The circular bacterial replicons are projected as horizontal lines with fixed length of 360, representing the degrees of a circle. A coordinate system is created with length of the replicon along the *x*-axis and the number of replicon used on the *y*-axis. When a query sequence matches with a gene/protein of a particular replicon, the BLAST-results are depicted as an “*x,y*” position in a specially adapted plot. This tool allows the visualization of the results from the whole data to a particular gene/protein in real time with low computational resources.

Thousands of completely sequenced bacterial and archaeal genomes are currently available on public repositories and this number is increasing rapidly. This information allows the accomplishment of exhaustive comparative genomic studies. One of the most widely used tools for searching sequence similarity is BLAST (Altschul *et al.*, 1990), available to run from several web servers or locally with a stand-alone version. Running BLAST from a web server is limited to comparing a small number of query sequences at the same time. When running a BLAST local-version it is possible to include as many query sequences as desired. Nevertheless, additional programming skills are required to extract information.

Genomic information is a powerful source for getting insight into microbial traits and functions. Considering the large quantity of inherent data, its study depends on bioinformatics tools. Genomic mining is used to find the genes or clusters of genes that code for a specific biological function in sequenced genomes. One of the most successful approaches to search for these genes is the use, as a query sequence, of either the most conserved gene/protein or of a representative one with known function. That result is then used as a starting point for scanning its genomic context to find the rest of the genes involved in the feature of study. That genomic context characterization usually involves BLAST or sequence alignment comparisons to forecast their functions. Nevertheless, that strategy is time consuming. Alternatively, performing a large-scale BLAST search allows the inclusion, as query, of multiple nucleotide or amino acid sequences of those genes/proteins. It saves time for bioinformatic characterization, but also generates many BLAST results that need to be analyzed.

Many biological processes are encoded in gene clusters, so multiple searches are required to determine the presence of a full biosynthetic pathway or bacterial operon. Tools to perform these searches in a single run, and to sort and display results in an easy way to analyze them are needed as well. Currently, several tools are available and quite useful to search single and multiple genes/proteins by sequence similarity (Fong *et al.* 2008; Revanna *et al.* 2009; Despalins *et al.* 2011; Medema *et al.* 2013; Neumann *et al.* 2014). These tools include flexible configuration options, but only display a limited number of data. Therefore, they require extra steps to fully browse through the results. Hence, the number of results that can be displayed simultaneously is limited.

In this study we introduced a platform-independent, free and open to use web tool (available at http://www.blast-xyplot-viewer.icuap.buap.mx) that allows multiple BLAST searches against whole-sequenced bacterial/archaea genomes, and a novel strategy for visualizing vast data. To display an extensive number of BLAST-results simultaneously, we used the advantages of an (*x,y*) plot (Figure 1). This tool can be used to search for the presence, completeness and distribution of single genes/proteins, operons or full biosynthetic pathways in a particular taxon or biological hierarchy, or even in all sequenced bacteria/archaea, in a single run.

**Figure 1:**
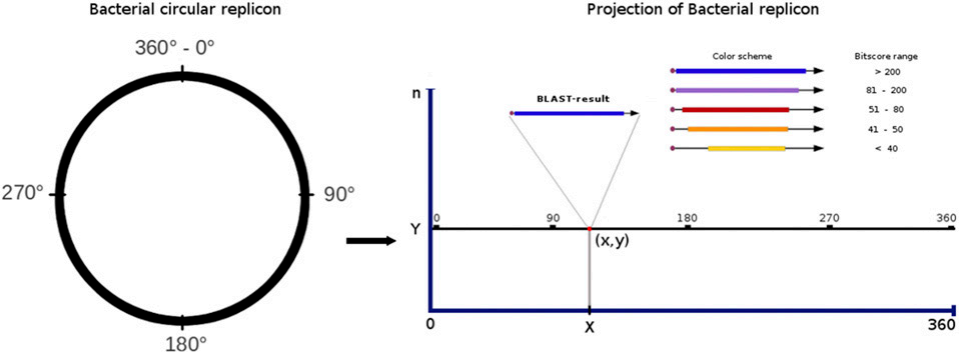
Linear projection of the circular bacterial replicon. The bacterial replicon is represented as a line with fixed length of 360 arbitrary units. An (*x,y*) plot scheme is used to depict BLAST-results where the *x*-axis represents the position of subject sequence into the bacterial replicon (with any value ranging from 0 to 360), and the *y*-axis corresponds to the number of replicons used in the search. See text for description.

## Materials And Methods

The visualization of massive BLAST-results uses an (*x,y*) dot plot scheme. To perform this representation, the circular bacterial replicons were projected as straight lines with fixed length equal to 360 (the degrees in a circle), providing a delimited space where any chromosome or plasmid can be represented independently of their actual size. In an (*x,y*) plot coordinate system, the *x*-axis represents the length of the replicons, and the *y*-axis represents the quantity of replicons (chromosomes or plasmids) used as database in the search. That number ranges from 1 (if only one replicon is used) to the maximum number of replicons used (*e.g.*, the chromosomes and plasmids of all sequenced bacteria/archaea). Using this projection, each BLAST-result is mapped by their *relative position* (0-360) instead of their real (nucleotide) position (Figure 1).

**Figure 2:**
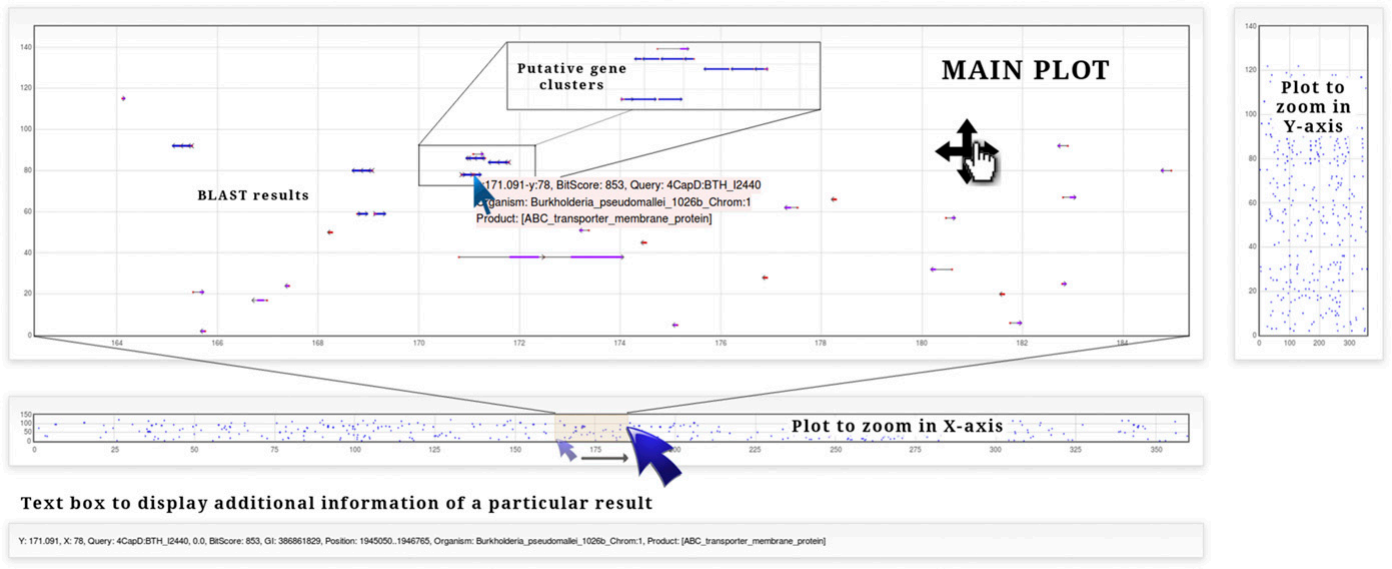
Screenshots of the plot used to display BLAST-results. Three plots and a text box are used to visualize data. The main plot allows zoom in/out, drag and displays information about the subject sequence on mouseover of BLAST-results. The two additional plots can be used to select a subrange of data in the *x*-axis (bottom plot) or in the *y*-axis (right plot). The text box displays additional information when clicking a particular BLAST-result.

When searching for a particular gene/protein in a genome(s) with BLAST, each result (subject sequence) is represented by a dot with an (*x,y*) coordinate. In addition to the dot, representing the origin of the subject gene, a vector with a magnitude proportional to its length, and with a direction according to the transcription sense is plotted. The BLAST-output (alignment between query and subject sequences) is represented by a thicker line over the vector, with a longitude proportional to the length of the region aligned and a color that depends on the significance defined by the BitScore value given by BLAST. That color varies from dark blue for the most significant to yellow for the least (Figure 1). The advantages of this visualization method are: 1) the interactivity of the plot, like zooming or dragging, can be used to visualize/analyze vast data from the whole results and focusing into a particular one in real time; 2) it is easily noticed if two or more BLAST-results are close to each other; and 3) BLAST-results that belong to many different replicons can be schematized simultaneously (Figure 2).

The input of BLAST-XYplot viewer is a set of sequences (or a single one) in FASTA format, including headers, that can be pasted in a text box or loaded as a file from the user’s computer, in plain text format. Users must type a Job title, provide a valid e-mail address and configure several parameters. BLAST searches can be performed against protein, genes or whole-genome bacterial/archaeal databases, depending of configuration setup. The BLAST Sequence comparison could be performed against genomes belonging to a particular genus (*e.g.*, *Escherichia*, *Enterococcus*, *Archaeoglobus*), a taxonomic group (*e.g.*, *Enterobacteriaceae*, *Proteobacteria*, *Euryarchaeota*) or all genomes. Currently, the genome list used by BLAST-XYplot viewer includes 5138 replicons, downloaded from the NCBI ftp server. Depending on the number of query sequences, databases used and jobs queue, the sequence comparison could take anywhere from a few minutes (search for less than twenty query sequences in a few genomes) to several hours (hundreds of query sequences in many genomes).

BLAST-results are recorded in a plain-text file in Json format and loaded into a specially adapted FLOT chart (http://www.flotcharts.org) to be visualized. In addition to the plot, a table of data in spreadsheet style allows users to sort and filter results (see http://www.blast-xyplot-viewer.icuap.buap.mx/tutorial for a graphical tutorial). The maximum number of data displayed on the web tool is limited to 50 thousand results because more than this quantity saturates the plot, hindering analysis. Nevertheless, the raw data file available for download could include up to one million BLAST-results.

The *Graph* results page consist of three plots and a text box (Figure 2). The main plot is used to visualize the BLAST results; it can be dragged and zoomed in or out. On mouseover of a particular result, a pop up text will appear with basic information about the result: relative position, BitScore value, query header, organism and gene product. By clicking on a particular result, additional information will appear on the text box below the plot. The two small plots allow users to zoom in on a particular region: on the *x*-axis, to visualize a section of all replicons; or in the *y*-axis to visualize whole replicons of a subgroup of microorganisms. To browse through results, a zoom could be applied using the small plot to select a range of around 25 degrees on the *x*-axis and drag the main plot with the left mouse button.

**Figure 3:**
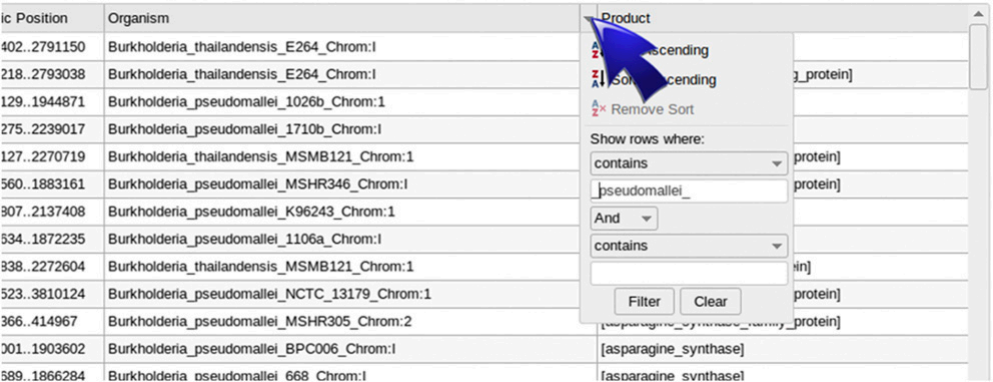
Table of data. The table lists BLAST-results in a spreadsheet format, and includes basic sort and filter options.

The *Table* page shows the BLAST-results in a basic spreadsheet. On mouseover of the header of a column, a small triangle will appear. By left clicking, a drop-down menu will be displayed to configure filtering or sorting options (Figure 3). After filter BLAST-results, data can be updated, in order to be visualized on the plot, by clicking on the “Actualize data” button bellow the table, and then “refresh” the *Graph* page.

The data can be downloaded from the *Graph* page as a Job-folder containing files with raw data (*_Sorted), filtered results (*_Sorted_Scored), *Table* and *Graph* in html format and their respective Json files. The raw file is a plain text and would be used for deeper scrutiny of the BLAST-result in the user’s computer.

The main advantages of this tool, when compared with other similar tools (Table 1), are the possibility to visualize several thousand BLAST-results simultaneously, highlighting *relative position* into the bacterial genome, and the interactivity of a plot that allows focusing on a particular result in real time using low computational resources. Additionally, this tool is capable of scanning many genomes simultaneously when dragging the plot through a zoomed portion of the *x*-axis, a feature not supported by any other tool.

**Table 1:** Comparison of XY-plot viewer with similar tools

	Large scale search	Precalculated results	Web Server	Display vast results simultaneously	Displays gene order	Real time zoom	Displays relative position in the genome	Displays gene context	Filters Data	Custom databases	Ref.
XYplot viewer	X		X	X	X	X	X		X	X	this work
PSAT	X	X	X		X		X	X		X	Fong *et al.* 2008
MultiGeneBlast	X				X			X		X	Mederma *et al.* 2013
BLASTgrabber	X			X					X	X	Neumann *et al.* 2014
Absynte			X		X			X		X	Despalins *et al.* 2011

### Data availability

The authors state that all data necessary for confirming the conclusions presented in the article are represented fully within the article. Supplemental material available at Figshare: https://doi.org/10.25387/g3.6288914.

## Results And Discussion

Case studies that can be performed easily with this tool include search for a single gene/protein, a small group of genes/proteins (small operon), a large operon or a full biosynthetic/degradative pathway, or multiple operons. All of these can be compared against a few, many or all of the sequenced genomes in the database. Some examples are depicted here (for an extended description see supplementary material).

One example of a small group of genes is the cluster that codes for Capistruin, a bacteriocin reported in *Burkholderia thailandensis* E264 and characterized by a knotted structure (Knappe *et al.* 2008). The Capistruin locus is composed by four genes (*capABCD*) that code for the bacteriocin precursor, two proteins involved in maturation and an exporter (Knappe *et al.* 2008). To search for this gene cluster in the remaining genomes of the *Burkholderia* genus, parameters can be defined as follows:

Job title: Search for Capistruin cluster in Burkholderia genomes

Sequence File: Capistruin.sec

Subject Data Base: Genus

Genus: Burkholderia

BlastType: Genes

E-value: 1e-08

MaxResults: 30000

A header for each sequences must be specified and an informative name is recommended, like those shown below:

>capA

ATGGTTCGACTTTTGGCGAAGC...

>capB

ATGCAACGGTCGCGCTATTTTC...

>capC

ATGGCGAAATCTATCGAACGCC...

>capD

ATGGCCCTTCCCATCCGAAACG...

**Figure 4:**
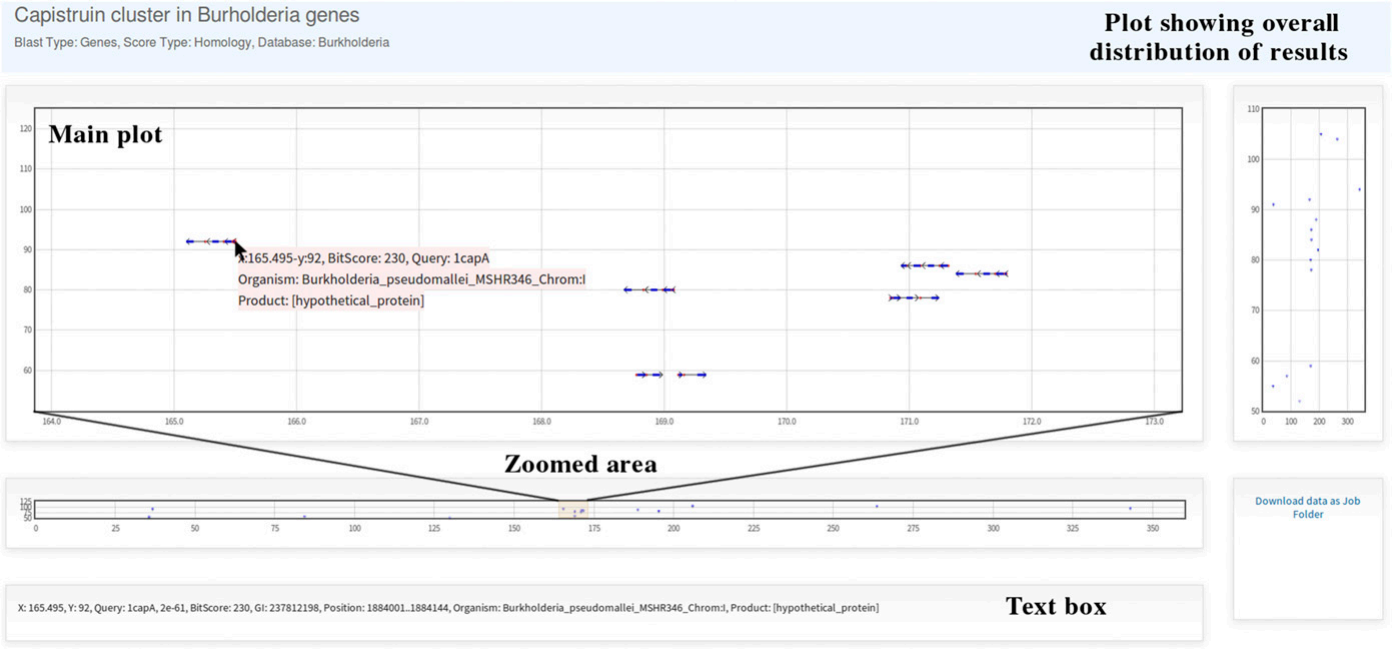
Plot of BLAST-results for Capistruin gene cluster search. The plot shows overall distribution of BLAST-results. By zooming data on the *x*-axis with the bottom plot, it is possible to see several clusters, and get information of a particular result when clicking on it.

The links to the *Graph* and *Table* pages are shown when the job is finished. If an e-mail address is provided, these links will be sent to it. In the *Graph* web page, the main plot shows the overall distribution of the BLAST-results. By selecting a region in the small plot, a range of data can be visualized more closely allowing the visualization of genes clustering (Figure 4).

**Figure 5:**
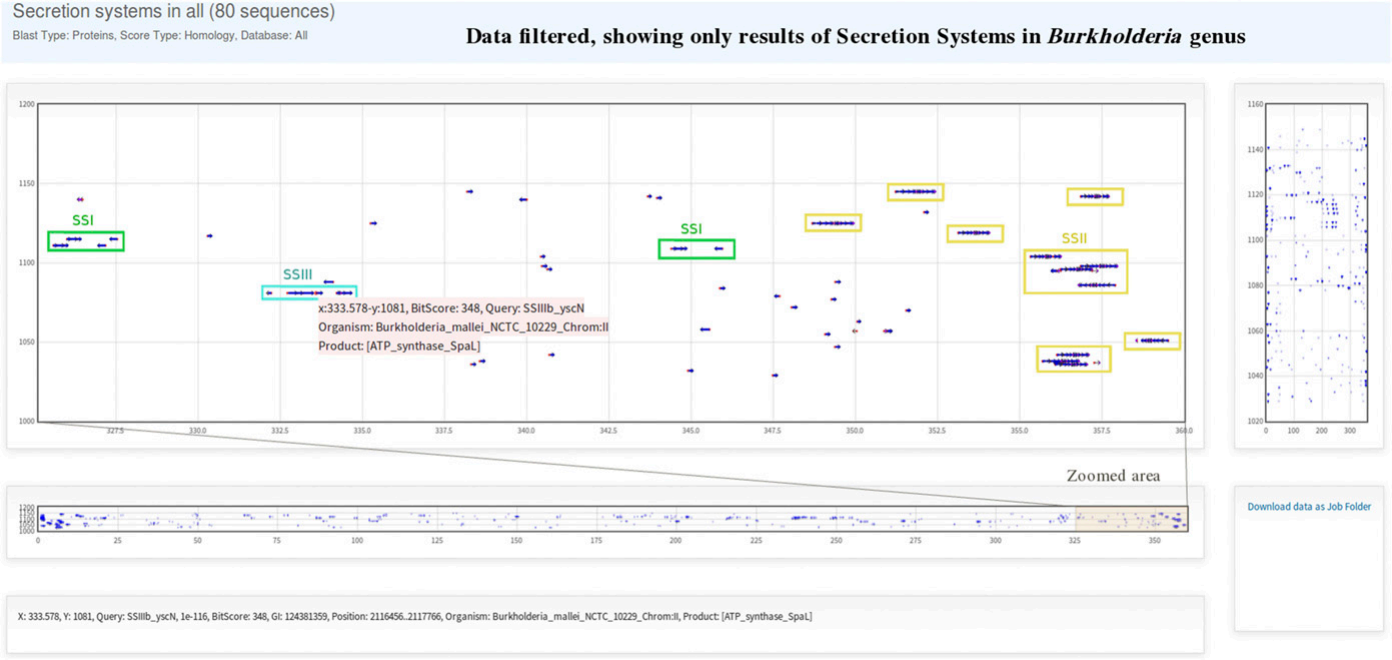
Plot of BLAST-results for Secretion Systems gene cluster search. The plot shows a section of the distribution of BLAST in a zoomed area from the degrees 325 to 360.

An example of a multiple operon case is the search of the six types of secretion systems. Here we used 80 sequences of representative secretion systems (Abby *et al.* 2016) and ran a BLAST search in all genomes. To browse through the huge amount of results generated by large jobs like this example, it may be more convenient to filter them by some criteria with the *Table*. To facilitate this process, the informative names of headers become relevant. The most useful way to name the sequence header could be: >SSI_HlyD, >SSI_TolC…, >SSII_GspC, >SSII_GspE…, and so on. By using this systematic nomenclature, it should be easy to filter results belonging to a specific type of secretion system. For example, to show only the results of the secretion system type I, we can type “SSI_” on the “contains” option of the drop down filter menu of the *Query* column in the *Table*, and then actualize results to be plotted in the *Graph*. Another option to filter results is to write the name of a desired organism in the drop down menu or a combination of both filter conditions. For example, to see the distribution of secretion systems in *Burkholderia*, data can be filtered by typing “Burkholderia_” in the *Organism* column and clicking on the “Actualize data” button to update the plot. When browsing the results it would be possible to find different kinds of gene clusters (Figure 5). Similarly, by filtering data by “SSII_” in the *Query* column, and by “gladioli_” in the *Organism* column the table will show only the type II secretion system in *B. gladioli*, and then can be displayed in the plot to see the distribution.

**Figure 6:**
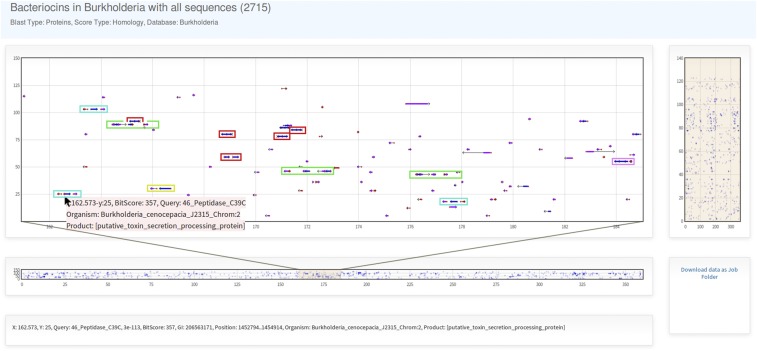
Plot of BLAST-results for the search of bacteriocins in *Burkholderia*. BLAST-results for more than 2700 query sequences are plotted together, highlighting different kind of putative bacteriocin gene cluster in the *Burkholderia* genus.

An example of multiple biosynthetic pathway searches may include all kinds of bacteriocin gene clusters. Bacteriocins are proteinaceous compounds synthesized by bacteria that inhibit the growth of other microorganisms. They are diverse in sequence, structure, mechanisms of action, and genetic regulation (Riley and Wertz 2002). This diversity makes laborious to find them by classical sequence similarity analysis. A strategy that has been used for finding them is to search for bacteriocin-related and conserved genes in a first step, and then browse the genomic context in order to find the non-conserved ones (Lee *et al.* 2008; Begley *et al.* 2009; Marsh *et al.* 2010; Wang *et al.* 2011; Singh and Sareen 2014). As an alternative of that strategy, we downloaded over 2700 sequences of bacteriocins and related proteins (*e.g.*, those related to maturation or exportation) from databases (Bru *et al.* 2005; Nikolskaya *et al.* 2006; Rawlings *et al.* 2006; Saier *et al.* 2006; Hammami *et al.* 2010; Marchler-Bauer *et al.* 2010; van Heel *et al.* 2013), and performed a large scale BLAST-search of bacteriocins in the *Burkholderia* genus. The BLAST-results plotted in the *Graph* highlights different putative bacteriocin gene cluster (Figure 6).

### Conclusion

The study of bacterial genomes using bioinformatic tools is becoming a more common strategy to understand microbial functions. Particularly, genome mining is used to find specific genes or clusters of genes in new sequenced genomes. The genomic characterization usually involves BLAST or sequence alignment comparison of genes/proteins to forecast their function. Performing a large scale BLAST search allows to include multiple query sequences of all genes/proteins involved in a particular biological function, in order to trace them in a single hit. This new strategy of data visualization by *relative position* provides a space where the enormous data generated by large-scale BLAST searches can be placed. This approach projects the data in an ordinate, interactive and intuitive format that is useful to handle and analyze vast genomic information simultaneously.
